# Investigation of airborne spread of COVID-19 using a hybrid agent-based model: a case study of the UK

**DOI:** 10.1098/rsos.230377

**Published:** 2023-07-26

**Authors:** Hafijur Rahaman, Debashis Barik

**Affiliations:** School of Chemistry, University of Hyderabad, Central University PO, Hyderabad 500046, Telangana, India

**Keywords:** COVID-19, airborne, diffusion, agent-based hybrid model, vaccination

## Abstract

Agent-based models have been proven to be quite useful in understanding and predicting the SARS-CoV-2 virus-originated COVID-19 infection. Person-to-person contact was considered as the main mechanism of viral transmission in these models. However, recent understanding has confirmed that airborne transmission is the main route to infection spread of COVID-19. We have developed a computationally efficient agent-based hybrid model to study the aerial propagation of the virus and subsequent spread of infection. We considered virus, a continuous variable, spreads diffusively in air and members of populations as discrete agents possessing one of the eight different states at a particular time. The transition from one state to another is probabilistic and age linked. Recognizing that population movement is a key aspect of infection spread, the model allows unbiased movement of agents. We benchmarked the model to recapture the temporal stochastic infection count data of the UK. The model investigates various key factors such as movement, infection susceptibility, new variants, recovery rate and duration, incubation period and vaccination on the infection propagation over time. Furthermore, the model was applied to capture the infection spread in Italy and France.

## 1. Introduction

Coronavirus disease 2019 (COVID-19) is an influenza-like disease with both pulmonary and non-pulmonary presentations and it is caused by acute respiratory syndrome coronavirus SARS-CoV-2 [1,2]. From the initial discovery in Wuhan, China in December 2019, the COVID-19 disease has rapidly spread all over world leading to the declaration of pandemic by the World Health Organization in the March of 2020. A significant number of the infected populations required hospitalizations and intensive medical interventions due to severe pulmonary complications. The disease has resulted an unprecedented public health crisis with nearly 76.7 million confirmed infections and 6.9 million deaths worldwide [3]. The severity of the viral infection is comparable to the deadly Spanish flu of the early twentieth century. The economic loss due to the global pandemic is enormous with potential far-reaching societal impact [4]. Various non-pharmaceutical interventions such as social distancing, use of face masks, frequent sanitizing and washing of hands [5] and lockdown measures [6] were implemented to contain the spread of infection. In the later stage, mRNA and antigen-based vaccinations helped reduce the infection spread [7] and very recently antiviral drug Paxlovid has been approved by the Food and Drug Administration and European Medicines Agency for mild to moderate COVID-19 infection [8,9]. However, even with those measures the infection continued to rise and fall with multiple peaks in many countries across the globe. Therefore, it is quite crucial to study and understand the dynamics of infection transmission from infected to healthy individuals.

Mathematical modelling has proven to be quite helpful in making strategic decisions by policy making bodies in limiting and mitigating infection spread. The main objective of mathematical modelling has been understanding the mechanisms of infection spread and subsequently underscoring effective non-pharmaceutical intervention guidelines [10,11]. Ordinary differential equation-based compartmental models [12] and agent-based models (ABMs) [13–15] are the two popular approaches of epidemiological investigation of infection spread over a large population. ABM is a detailed modelling methodology that includes granular details of heterogeneity in various epidemiological interactions among the individuals in a population. The compartmental models based on susceptible–exposed–infected–recovered (SEIR) [16–18] groups were used to determine the basic reproduction number of COVID-19 infection [19–21]. However, compartmental modelling cannot take into account person-to-person variabilities relevant to infection, transmission and recovery, spatial dimension of the epidemic spread and interventions. On the contrary, ABMs are capable of incorporating detailed social interactions and thus have become a useful tool in investigating infection spread due to smallpox [13], influenza virus [22], H5N1 influenza A [23], tuberculosis [24] and measles [25]. A number of epidemiological models of COVID-19 infection have been developed using agent-based modelling approach to investigate various non-pharmaceutical intervention strategies such as social distancing, usage of masks, contact tracing and subsequently these models also explored the spatial aspects of infection spread [26–33]. Particularly, it is used to investigate various levels of interaction details of the infection propagation in healthcare facilities [34,35], educational institutions [36–39], cities [40–42], states and countries [43–46].

In the ABMs, direct contact between the agents has been considered as the main mechanism of viral transmission from infected individual to healthy individual. However, in general, viruses are known to be transferred from one to another individual by inhaling virus-laden droplets and aerosols exhaled by an infected individual [47,48], contaminated surfaces [49] and also by contamination of fecal material [50]. While the virus-laden droplets travel in air a shorter distance, aerosols are known to travel a longer distance in air [51,52]. It is well established that tuberculosis, measles and chickenpox are transmitted via aerosol particles. Compelling evidences show SARS, MERS, influenza and smallpox are transmitted via aerial routes [53]. Reports suggested that close proximity in indoor places with poor ventilation such as restaurants [54,55], gyms [56], religious gatherings [57,58], public vehicles [59,60] has contributed to COVID-19 outbreaks. There are also compelling evidences of COVID-19 transmission at a longer distance among humans [61–63] and animals [64]. Speaking alone generates thousands of aerosol particles [65] and asymptomatic or presymptomatic individuals who do not cough or sneeze account for a large percentage of total infection (greater than 30%) [66]. More importantly COVID-19 viral transmission through touching surfaces is relatively small [67]. Therefore, aerial transmission occurs both in shorter range (less than 1–2 m) with shared breathing space and also in a longer range (greater than 2 m) via respiratory aerosols [68–70]. Finally, on 23 December 2021, World Health Organization declared that COVID-19 is indeed airborne in short range in an open environment and long-range transmission is possible in a closed environment.

An accurate model of COVID-19 thus must take into account airborne spread of the virus considering the updated understanding of virus transmission. Therefore, in the ABM framework, the viral transmission must include the spreading of virus-laden aerosols that leads to infection propagation among individuals who are in close contact with each other. Further a computationally efficient coarse-grained ABM capable of recapturing the temporal evolution of infection is also necessary. With these goals, we have investigated airborne spread and transmission of SARS-CoV-2 through diffusion in air in a hybrid discrete continuum (HDC) framework of ABM. We have developed an agent-based infection model where the SARS-CoV-2 virus was considered as continuous variable and spreads through the air by pure diffusion and the members of the human population were treated as discrete agents. The model was benchmarked against the daily infection count data of the United Kingdom (UK). The calculated daily infection count faithfully recaptures the normalized temporal stochastic profile and the normalized cumulative infection count of the UK. The model investigates various key factors such as movement, infection susceptibility, new variants, recovery rate and duration, incubation period and vaccination on the new infection counts of three age-based population groups. Furthermore, the model was applied to recapture the infection spread in Italy and France.

## 2. Material and methods

### 2.1. Model of infection

**Figure 1.**
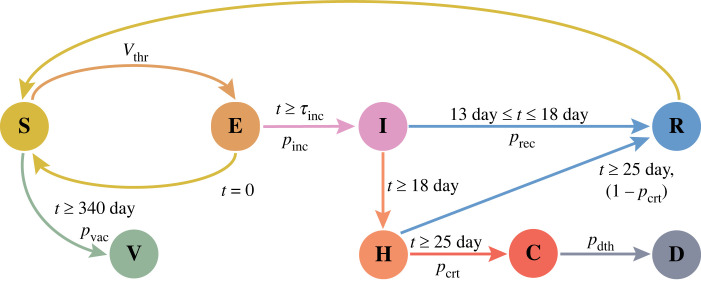
Schematic of hybrid model of infection transmission. An agent transitions from *susceptible* (S) to *exposed* (E) state upon crossing a threshold viral load (*V*_thr_) in air and the time is set to zero (*t* = 0). An *exposed* agent becomes *infectious* (I) with an age-linked probability of infection, *p*_inf_, after an incubation period (*τ*_inc_ = 6 days) or return to the original state. Recovery (R) from infection happens between day 13 and 18 with a probability of *p*_rec_ and beyond day 18 unrecovered infected agents are *hospitalized* (H). Beyond day 25, illness of some of the hospitalized agents may become *critical* (C) with a probability, *p*_crt_, and non-critical agents recover from illness. *Critical* agents may *die* (D) from the severity of the infection with an age-linked probability of *p*_dth_ and the surviving *critical* patients recover from the *hospitalized* state. Following the timeline of vaccination in UK, agents are *vaccinated* (V) with an age-linked vaccination probability *p*_vac_ post-day 340. The *recovered* individuals may also become reinfected with the SARS-CoV-2 after 90 day since recovery.

The hybrid model of infection considered three age-based population groups—child, young and old—with different characteristic probabilities of infection, death and movement. An individual in these groups is considered as a discrete agent possessing any one of the eight types of states at a particular time. These states are *susceptible* (S), virus *exposed* (E), virus infected or *infectious* (I), *recovered* from infection (R), *hospitalized* (H), *critical* (C) and *deceased* from infection (D) and *vaccinated* (V) (figure 1). A *susceptible* agent is a healthy individual without prior history of COVID-19 infection and its state changes to *exposed* state being exposed to a threshold amount of airborne virus (*V*_thr_). The *exposed* agent may transition to *infected* state with a characteristic age-group linked probability after an incubation period (*τ*_inc_). Following the literature, we have used an incubation period of 6 days for all age groups [1,71,72]. We used different values of infection probability based on the age groups considering that individuals in the older and child age groups are most and least vulnerable to SARS-CoV-2 viral infection, respectively [73]. The probabilities corresponding to old, young and child age groups follow the order *p*_inf,old_ > *p*_inf,yng_ > *p*_inf,chd_ [73–76].

During breathing, speaking, coughing and sneezing infected individuals produce virus-laden aerosols that diffuse through air and potentially infect other healthy individuals at a distance. In addition, the exposed population can also exhale virus with a smaller rate. We considered the air suspended virulent particles as continuous variable and its dynamics is represented by the reaction–diffusion equation as

2.1 $$\frac{dV}{dt}=\;k_{v,e}N_{e}+k_{v,i}N_{i}-\Gamma_{v}V+D_{v}\nabla^{2}V,$$

where *V* represents the concentration of virus in air with a diffusion coefficient *D_v_*. *k_v_*_,*i*_ and *k_v_*_,*e*_ are the rate constants of virus production by the infected and exposed individuals, respectively. The average lifetime (1/Γ*_v_*) of the virus in the air is given by the parameter Γ*_v_*. Both exposed and infected populations are potent in infecting others [77,78]; however, we have chosen higher rate of virus production by the infected individuals (*k_v_*_,*i*_ ≫ *k_v_*_,*e*_) considering asymptomatic individuals do not cough and sneeze [79]. The half-life of the virus (ln2/Γ_v_) was chosen to be 42 min which is shorter than the previously estimated half-life of approximately 65 min of SARS-CoV-2 in aerosols [80]. However, more recent literature has shown that the infectivity of the aerosolized SARS-CoV-2 decreases in a much faster time scale than previous estimation [81]. The diffusion coefficient of the virus (*D_v_* = 0.176 cm^2^ s^–1^) was chosen to be the same as that of the oxygen in air. The dimensionless equation for the virus is given by

2.2 $$\frac{d\tilde{V}}{d\tilde{t}}=\;\kappa_{v,e}N_{e}+\kappa_{v,i}N_{i}-\gamma_{v}\tilde{V}+d_{v}\nabla^{2}\tilde{V},$$

where the dimensionless virus and other parameters are given as $\tilde{V}=V/V_{0}$, $\tilde{t}=t/t_{0}$, *κ_v_*_,*e*_ = *t*_0_*k_v_*_,*e*_/*V*_0_, *κ_v_*_,*i*_ = *t*_0_*k_v_*_,*i*_/*V*_0_, *γ_v_* = *t*_0_Γ*_v_* and $d_{v}=t_{0}D_{v}/L_{0}^{2}$. The chosen characteristic time (*t*_0_) and length (*L*_0_) scales were 1 h and 2 km, respectively. With these values of *t*_0_ and *L*_0_, the values of dimensionless degradation (*γ_v_*) and diffusion constants (*d_v_*) become 1.0 and 1.58 × 10^–8^, respectively. In order to obtain a numerical solution of dimensionless equation, equation (2.2) was discretized using standard finite-difference method. On a two-dimensional lattice, *V* at the lattice point (*j*, *k*) and time (*l* + 1) is expressed as a function of *V* at the four adjacent lattice sites at time *l*. The discretized version of the equation is given as (dropping the ∼ notation)

2.3 $$V_{\;j,k}^{l+1}=(1-4\delta_{V}-\gamma_{v}\Delta t)V_{\;j,k}^{l}+\delta_{V}(V_{\;j+1,k}^{l}+V_{\;j-1,k}^{l}+V_{\;j,k+1}^{l}+V_{\;j,k-1}^{l})+(\kappa_{v,e}{N_{e}}_{\;j,k}^{l}+\kappa_{v,i}{N_{i}}_{\;j,k}^{l})\Delta t,$$

where *δ_V_* = (*d_V_*Δ*t*)/Δ*x*^2^ with Δ*t* and Δ*x* representing step lengths of time and space assuming same spatial step lengths in both the lattice directions (Δ*x* = Δ*y*).

### 2.2. Model of recovery and death

The *infected* agents stay in the infected state at least for 7 days since the detection on day 6 [82]. Thus after 7 days since detection (or 13 days since contraction), an infected agent may recover from viral infection in a probabilistic manner. According to this model, an *infected* agent recovers from the infection on any day between day 13 and day 18 with a recovery probability, *p*_rec_. Therefore, the average infected phase becomes 9.5 days. Prolonged infection required hospitalization and on average COVID-19 infected patients stayed 7 days in hospital before recovery [83]. Therefore, the agents who did not recover on day 18 are now labelled as *hospitalized* (H) and stay in that state for 7 days. On day 25, some agents become critically ill with a probability of *p*_crt_ and the remaining agents recover from infection with a probability of (1 − *p*_crt_). The critically ill agents may recover or decease due to prolonged suffering from the illness. We have again implemented age-linked probability of death (*p*_dth,old_ > *p*_dth,yng_ > *p*_dth,chd_). With the implementation of the age-linked probabilistic model of death, a fraction of population does not survive and is counted as dead. The remaining fraction remains as critically ill and undergoes the steps of post day 25. Before 100 days and after 320 days, the death probabilities were highest and lowest, respectively. The *recovered* agents can become reinfected after 90 days since recovery.

### 2.3. Model of vaccination

**Figure 2.**
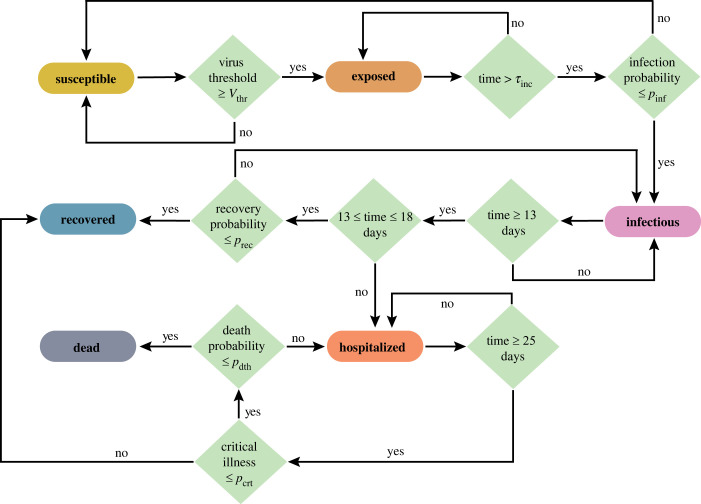
Flowchart of the hybrid model of infection spread and death. The vaccinations of the susceptible agents are not shown in the flowchart.

Various types of vaccines were developed against SARS-CoV-2, both mRNA and antigen based, to immunize populations of different age groups. In order to make the model more realistic and to study the effect of immunization on the infection propagation, we incorporated vaccination of agents following the timeline of vaccination in the UK. Vaccination of older population began in the first week of January 2021 in the UK. Considering that a complete vaccination requires two doses a month apart, in the model we initiated the vaccination from 1 February 2021 (day 340) without considering two separate doses. In the UK, vaccination started with the people in the old age group and then young and child age groups were immunized. We followed a similar vaccination strategy in the model. Older agents were vaccinated with a probability of *p*_vac_ in the first month, and *p*_vac_ was increased by 1.5 times for the rest of the period in the simulation. The vaccination probabilities for the younger agents were 0.5 × *p*_vac_, 0.75 × *p*_vac_ and *p*_vac_ in the first month, second month and beyond, respectively. The vaccination of the child population in the UK started from the middle of August 2021 and following that the vaccination of the child age groups was initiated from 15 August 2021 (day 535). In the model, the vaccination probability for the child was 0.5 × *p*_vac_ and *p*_vac_ for the first month and the rest of the duration, respectively. We assumed that the vaccinated agents are fully immune from COVID-19 infection and thus we did not consider any possibility of break-in cases. The flowchart in figure 2 summarizes the implementation of the entire model.

### 2.4. Model of movement

Movement of human population is a crucial aspect in the spread of COVID-19 infection. We introduced unbiased random movement of the agents on a two-dimensional lattice where an agent can jump to the one of the four adjacent sites of the square lattice with equal probabilities. In the unbiased random walk model, the probability of jumping to any one of the sites is *N*^−1^, where *N* is the number of available sites for the jump. In order to avoid directional bias, jump site was selected randomly when more than one adjacent sites are available. If all adjacent sites are occupied, then the agent does not move. For the old, young and child age groups we have introduced age-linked movement probability—*p*_mov,old_, *p*_mov,yng_ and *p*_mov,chd_, respectively. The probabilities of movement for the young and child populations were considered to be highest and lowest, respectively. The value of movement probabilities also depends on the health state where *susceptible* and *exposed* have highest and lowest movement probabilities, respectively. The *infectious* agents are not allowed to move considering the isolation state of the infected individuals. The *recovered* agents regain their movement corresponding to *susceptible* agents. It is important to note that there is a possibility that healthy agents may come to a close proximity of an infected agent and get exposed to the virus due to the unrestricted movement of the healthy agents. Thus even with complete restriction of movement of the infected agents, infection may still spread due to the movement of the healthy agents.

### 2.5. Simulation method

The health state of an agent was monitored and updated following the rules of infection, recovery, death and vaccination of over time across all lattice points. The viral loads on the lattice sites were determined by numerically solving the reaction–diffusion equation of the virus (equation (2.3)). The infection propagation was simulated on a two-dimensional square lattice having a dimension of 2 km × 2 km and the lattice was divided into 1000 × 1000 uniform grids with a grid size of 2 m (Δ*x* = 0.002). In the beginning of the simulation, we randomly distributed 90 000 *susceptible* agents uniformly in the entire lattice with 3 *infected* agents around the centre of the lattice. Fifty per cent of the population was young and the remaining 50% was equally distributed between children and older age groups. The integration time step was Δ*t* = 1.0 representing a time scale of 1 h. The parameters used in the model are listed in table 1.

**Table 1.** List of parameters and their values in the model. The diffusion constant and the half-life of the virus were taken from the literature. All other parameters were estimated to benchmark the model with the data from the UK.

parameter	description	value	unit
*d_v_*	diffusion coefficient of virus (0.176 cm^2^ s^–1^)	1.58 × 10^–8^	dimensionless
*t*_0_	characteristic time	1	h
*L*_0_	length of the lattice	2	km
*κ_v_*_,*i*_	virus production by the infected agent (5.3 h^–1^ person^–1^)	5.3	dimensionless
*κ_v_*_,*e*_	virus production by the exposed agent (3.57 h^–1^ person^–1^)	3.57	dimensionless
*γ_v_*	degradation of virus (1 h^–1^) [80,81]	1.0	dimensionless
*V*_thr_	threshold value of virus	0.014	dimensionless
*p*_mov,old_	movement probability of old	0.54	2 m^–1^ h^–1^
*p*_mov,yng_	movement probability of young	0.63	2 m^–1^ h^–1^
*p*_mov,chd_	movement probability of child	0.45	2 m^–1^ h^–1^
*p*_mov,exp_	movement probability of any exposed agent	0.225	2 m^–1^ h^–1^
*p*_rec_	probability of recovery	0.02	h^–1^
*p*_crt_	probability of critical condition	0.60	h^–1^
*p*_vac_	vaccination probability	0.003	h^–1^
*p*_inf,old_	infection probability of old	0.70	h^–1^
*p*_inf,yng_	infection probability of young	0.60	h^–1^
*p*_inf,chd_	infection probability of child	0.30	h^–1^
*τ*_inc_	incubation period	6	day
	*death probabilities until 100 days*		
*p*_dth,old_	death probability of old	0.90	h^–1^
*p*_dth,yng_	death probability of young	0.70	h^–1^
*p*_dth,chd_	death probability of child	0.30	h^–1^
	*death probabilities between 101 and 320 days*		
*p*_dth,old_	death probability of old	0.70	h^–1^
*p*_dth,yng_	death probability of young	0.50	h^–1^
*p*_dth,chd_	death probability of child	0.10	h^–1^
	*death probabilities between 321 and 625 days*		
*p*_dth,old_	death probability of old	0.20	h^–1^
*p*_dth,yng_	death probability of young	0.10	h^–1^
*p*_dth,chd_	death probability of child	0.05	h^–1^

An important aspect of the infection spread is lockdown that limits the movement of population. We used the lockdown pattern of the UK maintaining the timeline of various types of lockdowns during the course of the simulation. Specifically, in addition to the unrestricted movement phase (*Unrestricted*) of the pre-COVID period, we used three types of lockdowns—*Lockdown-1*, *Lockdown-2* and *Lockdown-3*. In the *Unrestricted* phase, *susceptible* and *exposed* agents move with their basal values of movement probabilities. *Lockdown-1* phase represents the full lockdown with only relaxation to essential services. *Lockdown-2* and *Lockdown-3* are partial lockdowns with moderate and greater relaxation to nonessential services, respectively. In *Lockdown-1*, *Lockdown-2* and *Lockdown-3*, the values of the movement probabilities were reduced by 67%, 55% and 22% of the *Unrestricted* probabilities. In table 2 types of lockdowns, their timeline and modification of the movement probability based on the state of lockdowns are listed for the UK. The ‘time zero' in the simulation corresponds to 26 February 2020. For statistical accuracy, we ran multiple simulations with different initial spatial distributions of agents and averaged the infection and death counts over 10 independent simulation runs. The model codes can be downloaded from https://github.com/dbarikUoH/Covid19Codes. A single simulation run typically takes about 30 min in a workstation containing two Intel Xeon Gold 6226R @2.90 GHz processors. The model was benchmarked with the infection data of the UK by trial-and-error tuning of parameters.

**Table 2.** Timeline of various types of lockdowns used in the model for the UK. Source: https://en.wikipedia.org/wiki/Timeline_of_the_COVID-19_pandemic_in_the_United_Kingdom.

timeline	measures	lockdown type	duration (day)	factor multiplied to movement probability
day 1–28 (26 February to 25 March 2020)	pre-pandemic	unrestricted	28	1.0
day 29–96 (26 March to 1 June 2020)	lockdown with relaxation to essential services	Lockdown-1	68	0.33
day 97–188 (2 June to 1 September 2020)	lockdown with relaxed restrictions	Lockdown-2	92	0.45
day 189–252 (2 September to 5 November 2020)	relaxed restrictions with open schools	Lockdown-3	64	0.78
Day 253–289 (6 November to 12 December 2020)	lockdown with relaxed restrictions	Lockdown-2	37	0.45
day 290–313 (13 December 2020 to 5 January 2021)	pre-pandemic	unrestricted	24	1.0
day 314–470 (6 January to 8 July 2021)	lockdown with relaxed restrictions	Lockdown-2	157	0.45
day 471–625 (9 July to 13 November 2021)	relaxed restrictions with open schools	Lockdown-3	155	0.78

### 2.6. Model benchmarking

With the focus of capturing the infection spread dynamics in the UK, we parametrized and benchmarked the hybrid model with the daily infection count data of the UK for 625 days starting from 26 February 2020. We implemented the timeline and type of lockdown in the UK by modifying the movement probabilities during the course of the simulation (table 2). Consistent with the vaccination of various age groups in the UK, we implemented vaccination of three age groups in the model (see Model of vaccination for details). The daily infection and death data of the UK are publicly available at the Government of UK website [84]. To study the infection spread among various age groups, we made comparison of temporal infection spread in the child, young and old age groups with the data of the same age groups in the UK. Age-based infection data are also available in the same database. For comparison, we created three age groups from the age demographic data of the UK. Specifically, we combined the infection counts of 0–14, 15–59 and 60–95 years of age and labelled them as child, young and old age groups. Furthermore, we determined the effect of various parameters on the infection spread. Particularly, the effects of movement and infection probabilities of various types of population, incubation period of the virus, duration of infected phase, vaccination rates and use of mask in the model were investigated. We calculated the infection spread in France and Italy by considering the timeline of lockdowns of these two countries (electronic supplementary material, tables S1 and S2). The daily infection data of these two countries were obtained from the World Health Organization website [3].

## 3. Results

### 3.1. Infection spread in the UK: comparison with daily infection counts

**Figure 3.**
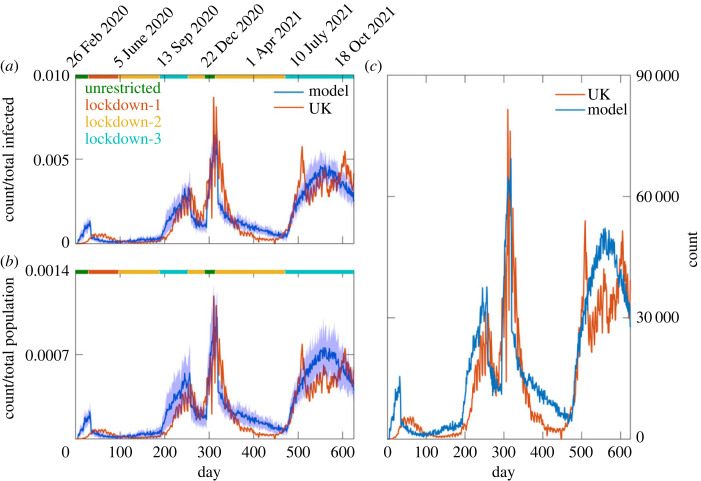
Comparison of model calculated normalized daily infection count with the normalized daily infection count of the UK (*a*,*b*). The model and UK data were normalized by their respective total infection counts until 625 days (*a*) and by their respective total populations (*b*). The shaded region in the model data indicates the ± standard deviation (s.d.) on the average data. The horizontal bars on the top of the plot represent various types of lockdowns that were implemented in the UK. The fractions of total population infected in the model and UK, respectively, are 0.165 and 0.137. The daily infection count in the model is multiplied by a scaling factor to compare with the infection data of the UK (*c*). The scaling factor was the ratio of total population of the UK (68 716 438) to the total population in the model (90 000).

In order to determine the number of new infections per day, we counted the total number of newly infected individuals, across all three age groups, during a period of 24 h over the entire lattice. Figure 3 shows the comparison of average number of new infections per day from the model with the daily infection data from the UK. For comparison, the model and the UK infection data were normalized by the respective total number of infections until 625 days (figure 3*a*) and also by the respective total number of population (figure 3*b*). Furthermore, we compared the actual counts of the infected agents in the model with the data of the UK by multiplying the model data with the ratio of the total population of the UK to the total population in the model and obtained a good agreement (figure 3*c*). The normalized 7-day running average infection count from the model agrees well with the UK data (electronic supplementary material, figure S1). The diffusion-based infection transmission model captures well the temporal stochastic evolution of daily infection count in the UK. The probabilistic natures of movement, infection, recovery and death are convoluted into the stochastic variations of number of new infections every day. The run-to-run variations can be noted from the plot of individual trajectories and also from the coefficient of variation (=standard deviation/mean) of the daily infection counts calculated over multiple runs (electronic supplementary material, figure S2). At the top of the plot, the horizontal bars indicate various types of lockdown phases. These bars indicate that there is a direct correlation between the increase of infection count with the higher movement of the population due to removal of lockdown. The infection count starts to decrease soon after the implementation of movement-limiting lockdown. Therefore, the movement of the population has a great role in determining the infection outbreak. The rapid infection outbreak around day 280, after the second peak, resulted the largest peak in the infection around day 310. Prior to the outbreak the infection count was significantly high as evident from the daily infection count during day 250–280. The significantly large pre-existing infection count and the partial reopening of the country during the holiday season of 2020 (table 2) both contributed to rapid outbreak during that period. In the summer of 2021, the delta variant of COVID-19 was found to be the dominating strain in the UK and the variant was found to be more contagious than the previous strain [85]. In order to take into account the effect of the delta variant, the infection probability was increased by 40% beyond day 480 across all age groups. Therefore, the lifting of lockdown measures and new variant of COVID-19 play a great role in the increase of new infection counts. Proximity of *infected* individuals to *susceptible* individuals is a key factor to the spread of infection through the aerial route. Although the *dead* and *recovered* agents spread across the lattice uniformly, the *exposed* and *infected* agents remain at the periphery due to the spread of the infection from the centre of the lattice (electronic supplementary material, figure S3).

**Figure 4.**
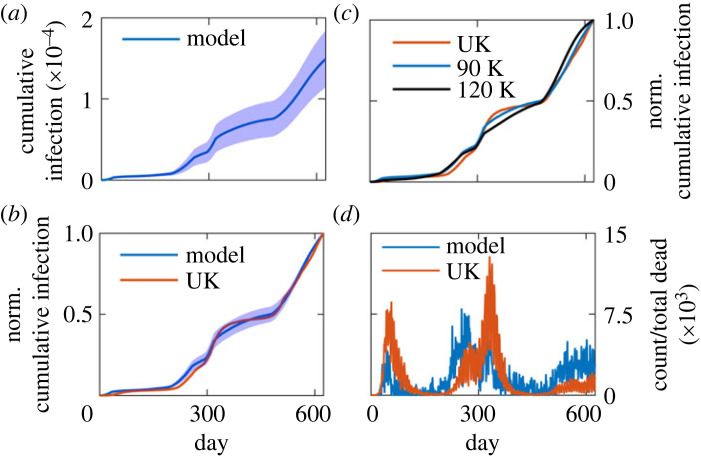
(*a*) Plot of model calculated cumulative infection count. The solid line and the shaded region indicate the average and ± s.d. The averaging was done over 10 independent trajectories. (*b*) Comparison of calculated normalized average cumulative infection count with the UK data. The shaded region indicates ± s.d. of average. (*c*) Model calculated normalized cumulative infection count for different number of total populations. (*d*) Comparison of normalized death count from the model with the data of the UK.

In figure 4*a*, the actual average count of cumulative infection is presented along with its standard deviation. The model calculated normalized average cumulative infection count compares quite well with the normalized cumulative infection count in the UK (figure 4*b*). The initial number of population can be a key factor in the spread of COVID-19 virus via aerial route. Thus, we calculated the infection spread with a larger number of total agents (120 000) to find a similar trend of infection (figure 4*c*). We compared the model calculated daily death counts with the death data of the UK. Although the pattern of the model calculated daily death counts is somewhat similar to the data from the UK, the agreement between the model and the UK data is limited (figure 4*d*). We also compared the age demographic death data from the model with the data of the UK (electronic supplementary material, figure S4). The agreement is fair only for the older age group. As mentioned before, the model considers a simple approach to death; however, in reality the death dynamics involves many aspects that includes heterogeneity of health conditions of patients, treatment regimens, care facility. Thus, an accurate modelling of death dynamics may require a more sophisticated modelling approach than we have adopted here.

### 3.2. Infection spread in the UK: spreading among various age groups

**Figure 5.**
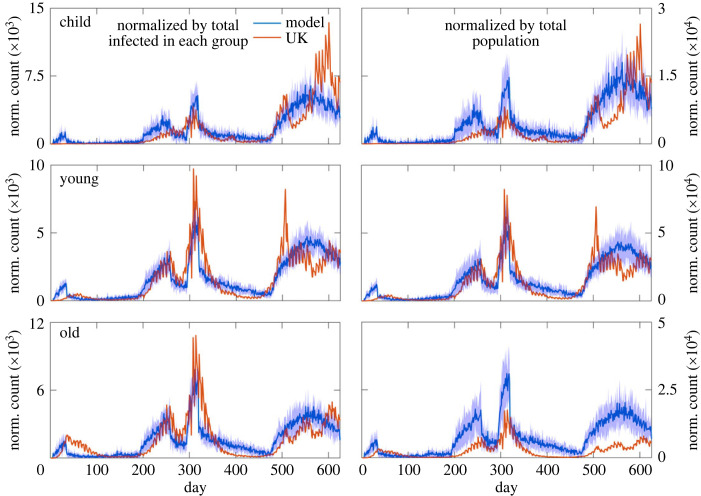
Comparison of normalized infection counts of three age groups of the model with those of the UK. The total populations in the model and UK were 90 000 and 68 716 438, respectively.

In order to determine the extent of infection spread among the three age groups, we compared the normalized infection counts of the three age groups of the model with the infection counts of the age groups in the UK. Figure 5 compares the infection counts of these age groups from the model and UK. In one set of comparison, we normalized the infection count of a particular group by the total number of infections in that group (figure 5, left panels). In another set of comparison, we normalized the infection data by the total population (figure 5, right panels). Across all three age groups the model data, normalized by their respective total count of infection, agree very well with the UK data. In the case of normalization by the total population, the agreement is fair. We have chosen 25:50:25 ratio of the population for the child, young and old age groups. However, careful consideration of the 2021 census data of the UK reveals that this ratio is 19:56:25 [86]. We verified that the infection counts remain nearly unaltered with the modified ratio of the population in the three age groups (electronic supplementary material, figure S5).

### 3.3. Infection spread in Italy and France: comparison with the model

**Figure 6.**
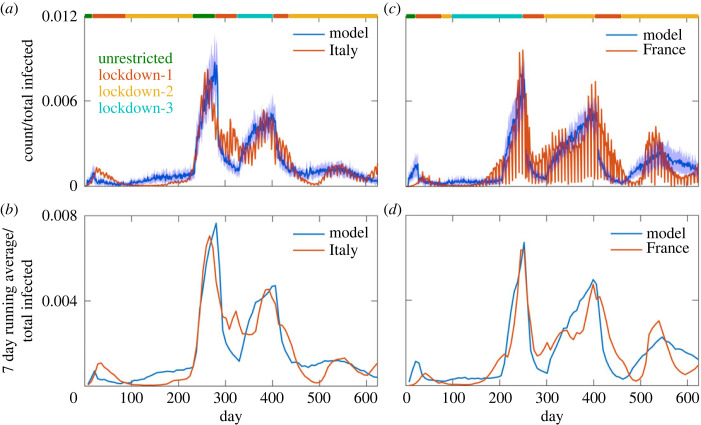
Comparison of model calculated average infection counts with the infection data of Italy (*a*,*b*) and France (*c*,*d*). The averaging was performed from ten independent trajectories and the shaded region indicates the ± standard deviation from the average. In (*b*,*d*), 7-day running averages are compared for the model and these two countries.

The model faithfully recaptures the temporal evolution of COVID-19 infection in the UK. However, to check the broader applicability of the model, we next used the model to determine the infection spread in Italy and France. We used the lockdown patterns in Italy and France based on the available information. We used the same set of parameters as used in the UK except for the factors that modify movement probability based on the lockdown types (electronic supplementary material, tables S1 and S2). In Italy, there were three strict public lockdowns and three somewhat relaxed lockdowns imposed during the first 625 days since 26 February 2020. The normalized infection counts from the model reasonably recapture the temporal profile of the infection counts in Italy (figure 6*a*). The rapid increase of infection around day 200 correlates well with the lifting of lockdown around that time and the decrease of the infection count correlates with the enforcement of lockdown. The normalized 7-day running average infection count from the model agrees well with the data (figure 6*b*). We then extended the infection spread calculations for France taking into account the lockdown patterns of France (figure 6*c*,*d*). Here too the increase and decrease of infection counts correlate will the implementation and removal of lockdown measures, respectively.

### 3.4. Parameter sensitivity analysis: effect of movement and infection rates

**Figure 7.**
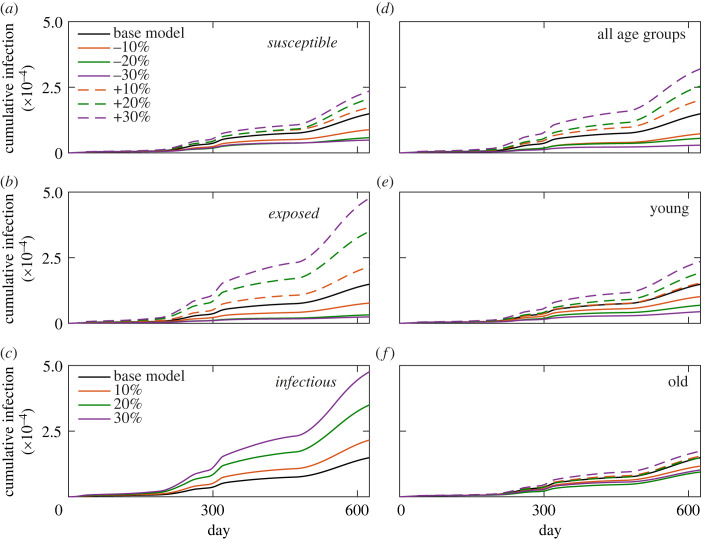
Effect of movement probabilities of the *susceptible* (*a*), *exposed* (*b*) and *infectious* (*c*) populations, by the indicated per cent, on the cumulative infection count. The effect of infection probability of all (*d*), young (*e*) and old (*f*) age groups on the cumulative infection count. All cumulative infection counts represent averaging over 10 independent trajectories.

We next determined the effects of individual parameters on the daily infection count by modifying a parameter from its base value for the UK model. As movement of population is an important factor in the spread of the infection, we modified the probability of movement across all three age groups and determined its effects on the daily infection count. The increase (or decrease) of movement of the *susceptible* population causes a systematic increase (or decrease) of infection counts (figure 7*a*). A 10% reduction of the movement probability across all age groups of the *susceptible* population does lead to a noticeable decrease in the infection count of the entire population. On the contrary, modification of movement probability of the *exposed* population results in a greater change in the infection counts (figure 7*b*). The systematic increase of infection counts with the increase of movement probability of the *exposed* population is more prominent from the early days. The model does not allow movement of the infected agents with the rationale that a diagnosis of COVID-19 infection would force the infected persons into isolation. However, if infected persons violate the isolation and move around then it contributes to rapid infection spread (figure 7*c*). Only with 10% movement probability of the *infectious* population would lead to a significant increase in new infection. Thus, the model suggests that limiting the movement of *exposed* population and complete isolation of *infectious* are important factors in containing the infection spread.

An *exposed* agent becomes infected in a probabilistic manner with an age-linked infection probability. To assess the effect of infection probability of the various age groups, we systematically modified the infection probability of various age groups. The infection count increases (or decreases) in a systematic manner with the increase (or decrease) of infection probability across all age groups (figure 7*d*). We then looked at vulnerability of individual age groups by altering their infection probability. Modification of infection probability of the younger age group results in stronger effects on the overall infection counts (figure 7*e*) when compared with the older age group (figure 7*f*). We found a milder effects of such modifications on the child age group (not shown). Although younger population have moderate infection probability, due to the larger population share (50%) and higher movement probability the effect of infection probability of the younger group is stronger when compared with the older group. These point out that new infection is a cumulative effect of factors such as age groups, infection susceptibility and movement. Although the severity of the infection among the younger population would be less, they have greater possibility of spreading the infection among the entire population.

### 3.5. Parameter sensitivity analysis: effect of incubation period, duration of infection and vaccination

**Figure 8.**
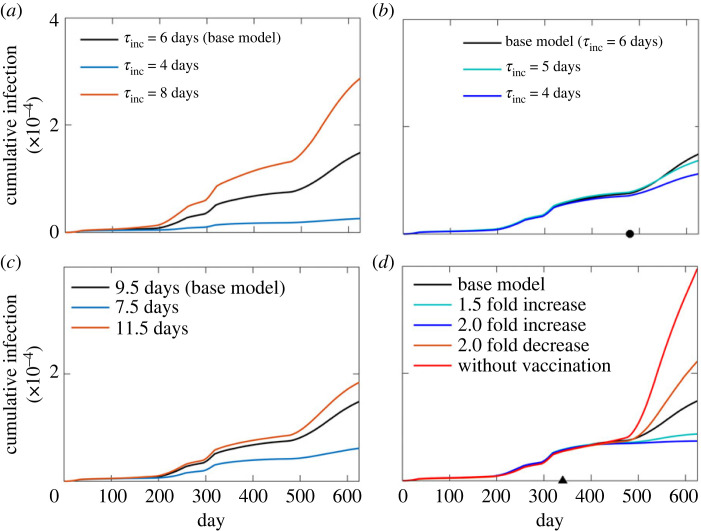
(*a*) The effect of different values of the incubation period (τ_inc_) during the entire course of simulation. (*b*) The impact of shorter incubation period of the delta variant on the infection spread post day 480 (indicated by the circle on the day axis). (*c*) The dependence of the duration of infected phase on the infection spread. (*d*) The variation of infection counts with the modification of the vaccination probability initiated on day 340 (indicated by the triangle on the day axis).

We further determined the effect of the incubation period (τ_inc_) on the infection spread (figure 8*a*). In the base model during the entire course of simulation, we used 6 days of incubation period. However, variation of the incubation period is known to occur across the globe [1,71,72,87]. Therefore, to study the effect of incubation period globally, we chose different values of τ_inc_ during the entire course of simulation and determined its effect on the infection spread. The model predicts that the infection count would be significantly less with a shorter τ_inc_ (4 days) when compared with a longer *τ*_inc_ (8 days). The results from figure 7*b* indicate that the movement of *exposed* population has a significant effect on the infection spread. Thus, a shorter incubation period leads to shorter ‘production' and ‘movement' phases of the *exposed* agents thereby resulting in reduction of the infection spread. Delta variant reportedly has shorter incubation period when compared with the earlier variants (B.1.177 and B.1.1.7) of SARS-CoV-2 [87]. Thus, to study the effect of τ_inc_ during the outbreak of delta variant, we reduced τ_inc_ from 6 days to 5 and 4 days post-day 480 (figure 8*b*). The cumulative infection count becomes less with shorter incubation periods post day 480. With shorter incubation period the duration of virus production by the exposed agents reduces and also the movement of infected agents gets restricted earlier due to early detection when compared with the situation with longer incubation period. The combined effects of these two lead to a reduction in the infection spread.

Next, we determined the effect of infected phase by altering the onset of recovery from the disease. We kept the total duration of recovery period fixed at 5 days while shifting the onset of recovery. In the base model, the average duration of the infected phase is 9.5 days. Advancing the onset of recovery by 2 days reduces the overall cumulative infection counts significantly and with longer infected phase the cumulative infection counts increase noticeably (figure 8*c*). Early recovery reduces the production phase of the virus, therefore limiting the aerial spread and subsequent contraction of virus by the *susceptible* agents. We found that modification of the recovery probability did not alter the infection count significantly (electronic supplementary material, figure S6).

Vaccination of the susceptible population is an integral part of pharmaceutical intervention against COVID-19. In the UK, the vaccination started in the beginning of 2021 and maintaining this timeline we initiated vaccination from the day 340 in the model. To determine the effect of the vaccination rate on the infection spread, we modified the vaccination probability (*p*_vac_) across all age groups keeping the timeline of the vaccination for various age groups unchanged. A systemic decrease and increase of cumulative infection count are noted with the systemic increase and decrease in vaccination probability, respectively (figure 8*d*). In order to determine the effect of not vaccinating, we set *p*_vac_ = 0 and determined the infection spread. Without vaccination the daily infection count would increase by multiple fold and with large increase in the cumulative infection count. Note that although the vaccination probability was set to zero on day 340, the major manifestation of not vaccinating in the infection count appears after day 480. The outbreak of the delta variant after day 480 (the summer of 2021; 20 June 2021) and the removal of lockdown around the same time would have resulted in a rapid increase of infection without vaccination.

### 3.6. Parameter sensitivity analysis: effect of mask usage

**Figure 9.**
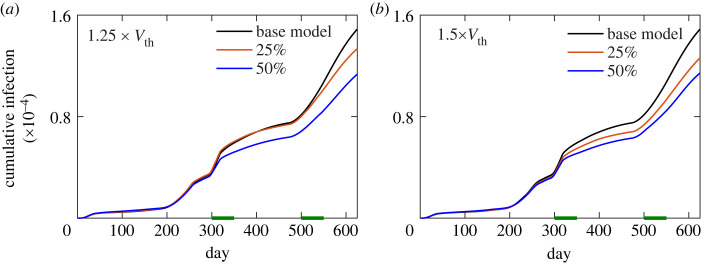
The effect of mask use by the indicated percentage of population during the third (day 300–350) and fourth (day 500–550) peaks of the infection spread in the UK. The mask use is implemented by increasing *V*_thr_ by 25% (*a*) and 50% (*b*). The durations of the mask use are represented by the horizontal green bars.

Behavioural aspects play a key role in the spread of any infectious disease. Certainly, in the case of COVID-19 wearing masks, washing and sanitizing hands frequently, and keeping distance from another person are crucial behavioural components of infection spread. The hybrid model does not consider many of these behavioural aspects explicitly. However, the effect of the wearing of masks is considered implicitly in the virus threshold parameter, *V*_thr_. *V*_thr_ determines the viral load required to cause an infection and therefore increased value of *V*_thr_ mimics wearing of masks. We implemented mask usage during an infection outbreak, by increasing *V*_thr_ for a certain fraction of population for a particular duration of time. We increased *V*_thr_ between day 300–350 and day 500–550 corresponding to the third and fourth peaks of the infection outbreak in the UK, respectively. The model shows a significant reduction in infection spread possible if 50% of the population uses masks that increases the *V*_thr_ by 25% (figure 9*a*). With a more effective mask with 50% increased *V*_thr_, a systematic decrease of infection spread is possible with the percentage of the population using masks (figure 9*b*).

## 4. Discussion

We have developed a coarse-grained hybrid model to study the aerial propagation of the COVID-19 virus and subsequent spread of infection on a two-dimensional lattice. The model was benchmarked against the daily infection count data of the UK and extended to Italy and France. The calculated daily infection count quite faithfully recaptures the normalized temporal stochastic profile of infection count for a period of 625 days since 26 February 2020. The accuracy of the model emphasizes the key role of aerial route of virus spread and consequent infection propagation from infected individuals to healthy individuals. Parameter sensitivity analysis reveals that movement of all types of individuals is a key factor in the spread of the infection. Particularly increased movement of exposed and infected individuals would cause a significant increase in the infection count. The model suggests that a shorter incubation period leads to a significant decrease in the infection count. It also represents that early detection of the infection is key to limit infection spread. Perturbations of infection probability suggest that infection spread among the younger population contributes to a greater extent in the overall infection count compared with old and child age groups. The greater share of younger population with higher movement probability are responsible for greater sensitivity of infection probability of the younger population. Therefore, in containing the infection, it is very important to take measures that limit infection spread among younger age groups. The model predicts that without vaccination there would have been more than three-fold increase in the cumulative infection count.

A large number of ABMs have been developed to investigate epidemiological aspects of COVID-19 infection and also to develop intervention strategies, both non-pharmaceutical and pharmaceutical, to control the infection spread. The spatial and temporal scales of these models varied considerably. These models studied the infection spread over a variety of geographical locations ranging from a large building (school or hospital) to a country and the temporal scales of the modelling outcomes range from a couple of weeks to several months [34–46]. Agent-based modelling tools such as Covasim [29] and PanSim [33] have been used to model infection spread in cities and countries over a period of a year. The main difference between our model and the contact-based ABMs is that we considered viral transmission via aerial route which is now accepted as the dominant mechanism of virus spreading. We have implemented a hybrid method of infection spread within the framework of ABM. Previous ABMs of COVID-19 mostly considered direct contact-based infection transmission. In addition, the hybrid model recaptures the infection spread over a long period of time (approx. 21 months).

The hybrid model recaptures the temporal dynamics of infection spread in the UK among various age groups over a period of nearly 21 months. However, the model has notable limitations particularly in the context of mechanism of the infection spread. It does not consider various subgroups of infected populations such as symptomatic, pre-symptomatic and asymptomatic separately. The contribution of asymptomatic spread of infection, particularly by the younger population, can be notable. We have not taken into account other less dominant avenues of viral transmission such direct contact and fomite transmission [88]. The model was parametrized with a finite number of agents representing the entire population of a country and thus a more realistic number of agents would certainly improve the applicability of the model. The hybrid model does not consider many behavioural aspects of population explicitly. For example, washing and sanitizing hands, being in close proximity, and wearing masks are not considered explicitly in the model. Nonetheless, the effect of the wearing of masks is considered implicitly in the virus threshold parameter, *V*_thr_. In the hybrid model the entire population is uniformly distributed throughout the lattice devoid of any geographical heterogeneity. However, in every country the population density is not uniform and densely populated areas are prone to rapid infection spread. Thus, the hybrid model can be extended to take into account population density heterogeneity. The model deals with only random movement in the local neighbourhood and does not consider moderate and long-distance travel. As the model is coarse-grained in nature it does not investigate location specific various micro-level interactions that occur in schools, education institutions, markets, banks etc. Therefore, a more comprehensive understanding and application of the hybrid model need further considerations of these factors.

## Data Availability

The model codes can be downloaded from a publicly available repository: https://github.com/dbarikUoH/Covid19Codes. The infection model data can be downloaded from https://github.com/dbarikUoH/Covid19Codes. The data are provided in electronic supplementary material [89].
